# 1,5-Bis(2-hy­droxy-3-meth­oxy­benzyl­idene)carbonohydrazide methanol 0.47-solvate

**DOI:** 10.1107/S1600536814004802

**Published:** 2014-03-12

**Authors:** Mouhamadou Moustapha Sow, Ousmane Diouf, Matar Seck, Aliou Hamady Barry, Mohamed Gaye

**Affiliations:** a Département de Chimie, Faculté des Sciences et Techniques, Université Cheikh Anta Diop, Dakar, Senegal; b Département de Chimie, Faculté de Medecine, de Pharmacie et d’Odonto-stomatologie, Université Cheikh Anta Diop, Dakar, Senegal; c Département de Chimie, Faculté des Sciences et Techniques, Université de Nouakchott, Mauritania

## Abstract

In the title compound, C_17_H_18_N_4_O_5_·0.47CH_3_OH, the virtually planar (r.m.s. deviation = 0.128 Å) carbonohydrazide mol­ecule is located on a twofold axis and conformation of its C=N bonds is *E*. There are short intra­molecular O—H⋯N hydrogen bonds between the hy­droxy groups and hydrazide N atoms. In the crystal, bifurcated N—H⋯(O,O) hydrogen bonds assemble the carbonohydrazide mol­ecules into a three-dimensional network. There are *C*_2_ symmetric voids in this network, 47% of which are occupied by disordered methanol mol­ecules.

## Related literature   

For related structures, see: Du & Zhang (2010 ▶); He *et al.* (2010 ▶); Kong *et al.* (2010 ▶). For the biological activity of carbonohydrazides, see: Bacchi *et al.* (1999 ▶); El-Gammal *et al.* (2012 ▶).
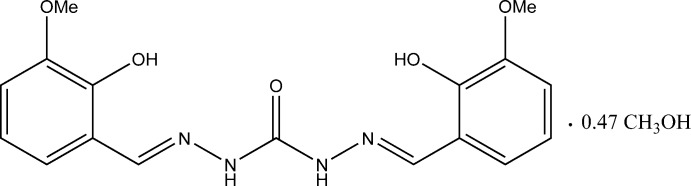


## Experimental   

### 

#### Crystal data  

C_17_H_18_N_4_O_5_·0.47CH_4_O
*M* *_r_* = 373.40
Orthorhombic, 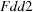

*a* = 9.4470 (7) Å
*b* = 17.5850 (9) Å
*c* = 22.8714 (12) Å
*V* = 3799.5 (4) Å^3^
*Z* = 8
Mo *K*α radiation
μ = 0.10 mm^−1^
*T* = 293 K
0.1 × 0.08 × 0.05 mm

#### Data collection  

Enraf–Nonius CAD-4 diffractometer
9573 measured reflections
862 independent reflections
658 reflections with *I* > 2σ(*I*)
*R*_int_ = 0.105
2 standard reflections every 120 min intensity decay: 2%

#### Refinement  

*R*[*F* ^2^ > 2σ(*F* ^2^)] = 0.044
*wR*(*F* ^2^) = 0.111
*S* = 1.25
862 reflections
146 parameters
1 restraint
H atoms treated by a mixture of independent and constrained refinement
Δρ_max_ = 0.17 e Å^−3^
Δρ_min_ = −0.19 e Å^−3^

### 

Data collection: *CAD-4 EXPRESS* (Enraf–Nonius, 1994 ▶); cell refinement: *CAD-4 EXPRESS*; data reduction: *XCAD4* (Harms & Wocadlo, 1995 ▶); program(s) used to solve structure: *SHELXS97* (Sheldrick,2008 ▶); program(s) used to refine structure: *SHELXL97* (Sheldrick, 2008 ▶); molecular graphics: *ORTEP-3 for Windows* (Farrugia, 2012 ▶); software used to prepare material for publication: *SHELXL97*.

## Supplementary Material

Crystal structure: contains datablock(s) I, global. DOI: 10.1107/S1600536814004802/gk2603sup1.cif

Structure factors: contains datablock(s) I. DOI: 10.1107/S1600536814004802/gk2603Isup2.hkl

Supporting information file. DOI: 10.1107/S1600536814004802/gk2603Isup3.cml

CCDC reference: 989432

Additional supporting information:  crystallographic information; 3D view; checkCIF report

## Figures and Tables

**Table 1:** Hydrogen-bond geometry (Å, °)

*D*—H⋯*A*	*D*—H	H⋯*A*	*D*⋯*A*	*D*—H⋯*A*
O1—H1*O*⋯N1	0.91 (5)	1.86 (5)	2.703 (5)	152 (5)
N2—H2*N*⋯O3^i^	0.94 (6)	2.38 (5)	3.044 (6)	128 (4)
N2—H2*N*⋯O1^i^	0.94 (6)	2.33 (6)	3.204 (6)	155 (4)

Symmetry code: (i) 
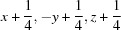
.

**Fig. 1.**
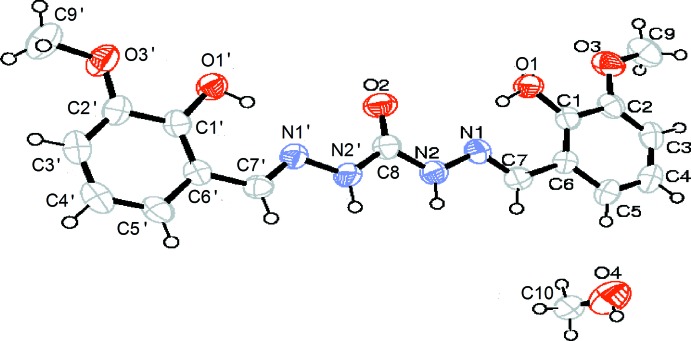
An ORTEP view of the title compound, showing the atom-numbering scheme. Displacement ellipsoids are plotted at the 50% probability level. Only one position of the disordered solvent methanol molecule is shown for clarity. The symmetry code for generating primed atoms is 2-x,-y,z

**Fig. 2.**
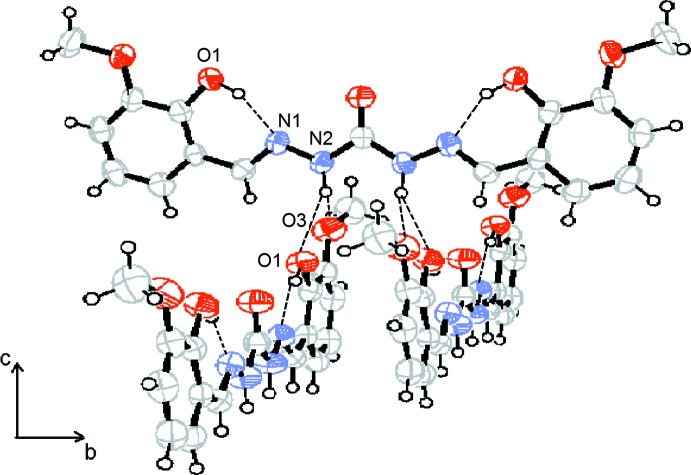
Intramoleculr and intermolecular hydrogen bonds. Solvent methanol molecules are omitted as they do not form hydrogen bonds.

## supplementary crystallographic information

### 1. Comment

Carbonohydrazide derivatives give rise to a large spectrum of biological properties such as antioxidant (El-Gammal *et al.*, 2012) and anticancer activities (Bacchi *et al.*, 1999). We report here the crystal structure of the title compound synthesized according to literature (He *et al.*, 2010; Du *et al.*, 2010). All parameters are within normal ranges and comparable with the related structures (Kong *et al.*, 2010).

The molecular structure of the title compound is shown in Fig. 1. The complete carbonohydrazide molecule is generated by a twofold crystallographic axis passing throuth the atoms C8 and O2. A three-center O···(N)H···O intermolecular hydrogen bond involving the amido H atoms and the phenoxo and methoxy O atoms is observed (Fig. 2). There are voids in a three dimensional network containing solvent methanol molecules. Only one methanol molecule can be accommodated in a small void that has C_2_ symmetry. This leads to disorder of methanol molecules. In addition refinement of occupancy factors of methanol O and C atoms converged at 0.234 (1), indicating that 47% of voids are occupied by the solvent.

### 2. Experimental

In a round bottomed flask, carbonohydrazide (1.0 g, 11.11 mmol) was introduced with methanol (10 ml). *o*-Vanillin (3.3 g, 22.22 mmol) dissolved in 10 ml of the same solvent was added. Two drops of glacial acetic acid were added while stirring. After one hour under reflux, the precipitate formed that after cooling to room temperature was filtered off and washed with cold methanol. The resulting solid was dried in air. The filtrate was left at room temperature. Slow evaporation of the solvent gave colorless crystals after one day. Yield: 95%; m.p. 378 K. Anal. Calc. for [C_17_H_18_N_4_O_5_] (%): C, 56.98; H, 5.06, N, 15.63. Found: C, 56.96; H, 5.04; N, 15.60. Selected IR data (cm^-1^, KBr pellet): 3291, 2942, 1696, 1553, 1200, 1167. ^1^H-NMR (DMSO-d6) δ: 3.8 (s, 6H, O—CH3); 6.7 – 7.1 (m, 6H, H_Aromatic_); 8.5 (s, 2H, H—C═N); 7.3 (s, 1H, H—N); 11 (s, 2H, H—O) p.p.m. ^13^C-NMR (DMSO-d_6_) d: 151.8 (C═O); 147.8, 146.1, 119.5, 119.4, 118.8, 112.8 (C_Aromatic_); 58,7 (–O—CH_3_).

### 3. Refinement

H atoms of the NH and OH groups were located in the Fourier difference maps and refined without restraints. Otherg H atoms were geometrically optimized and refined as riding on their carriers with *U*_iso_(H) = 1.2*U*_eq_(C)(1.5 for CH_3_ groups). Considerable disorder was detected for the solvent methanol molecule. The occupancy factor of the C and O atoms of methanol refined at 0.234 (1). Thus, there are 0.46 methanol molecules per one carbonohydrazide molecule in the crystal. Owing to a negligible anomalous dispersion effect the Friedel pairs were merged and the absolute structure was not determined.

### Crystal data

**Table:**

C_17_H_18_N_4_O_5_·0.47CH_4_O	*F*(000) = 1571.4
*M**_r_* = 373.40	*D*_x_ = 1.306 Mg m^−^^3^
Orthorhombic, *F**d**d*2	Mo *K*α radiation, λ = 0.71073 Å
Hall symbol: F 2 -2d	Cell parameters from 25 reflections
*a* = 9.4470 (7) Å	θ = 11–15°
*b* = 17.5850 (9) Å	µ = 0.10 mm^−^^1^
*c* = 22.8714 (12) Å	*T* = 293 K
*V* = 3799.5 (4) Å^3^	Prismatic, colorless
*Z* = 8	0.1 × 0.08 × 0.05 mm

### Data collection

**Table:**

Enraf–Nonius CAD-4 diffractometer	*R*_int_ = 0.105
Radiation source: fine-focus sealed tube	θ_max_ = 25.0°, θ_min_ = 2.6°
Graphite monochromator	*h* = −11→11
non–profiled ω/2θ scans	*k* = −1→20
9573 measured reflections	*l* = −27→27
862 independent reflections	2 standard reflections every 120 min
658 reflections with *I* > 2σ(*I*)	intensity decay: 2%

### Refinement

**Table:**

Refinement on *F*^2^	Primary atom site location: structure-invariant direct methods
Least-squares matrix: full	Secondary atom site location: difference Fourier map
*R*[*F*^2^ > 2σ(*F*^2^)] = 0.044	Hydrogen site location: inferred from neighbouring sites
*wR*(*F*^2^) = 0.111	H atoms treated by a mixture of independent and constrained refinement
*S* = 1.25	*w* = 1/[σ^2^(*F*_o_^2^) + (0.0306*P*)^2^ + 5.2614*P*] where *P* = (*F*_o_^2^ + 2*F*_c_^2^)/3
862 reflections	(Δ/σ)_max_ < 0.001
146 parameters	Δρ_max_ = 0.17 e Å^−^^3^
1 restraint	Δρ_min_ = −0.19 e Å^−^^3^

### Special details

**Table:**

Geometry. All e.s.d.'s (except the e.s.d. in the dihedral angle between two l.s. planes) are estimated using the full covariance matrix. The cell e.s.d.'s are taken into account individually in the estimation of e.s.d.'s in distances, angles and torsion angles; correlations between e.s.d.'s in cell parameters are only used when they are defined by crystal symmetry. An approximate (isotropic) treatment of cell e.s.d.'s is used for estimating e.s.d.'s involving l.s. planes.
Refinement. Refinement of *F*^2^ against ALL reflections. The weighted *R*-factor *wR* and goodness of fit *S* are based on *F*^2^, conventional *R*-factors *R* are based on *F*, with *F* set to zero for negative *F*^2^. The threshold expression of *F*^2^ > σ(*F*^2^) is used only for calculating *R*-factors(gt) *etc*. and is not relevant to the choice of reflections for refinement. *R*-factors based on *F*^2^ are statistically about twice as large as those based on *F*, and *R*- factors based on ALL data will be even larger.

### Fractional atomic coordinates and isotropic or equivalent isotropic displacement parameters (Å^2^)

**Table:**

	*x*	*y*	*z*	*U*_iso_*/*U*_eq_	Occ. (<1)
O1	0.6805 (4)	0.1635 (2)	0.02775 (15)	0.0559 (10)	
O2	1.0000	0.0000	0.0525 (2)	0.0641 (14)	
O3	0.4920 (4)	0.2637 (2)	−0.00424 (17)	0.0680 (12)	
N1	0.8123 (4)	0.0896 (2)	0.11591 (18)	0.0471 (10)	
N2	0.9127 (5)	0.0436 (2)	0.1418 (2)	0.0533 (11)	
C1	0.6048 (5)	0.1974 (3)	0.0728 (2)	0.0459 (12)	
C2	0.5016 (5)	0.2512 (3)	0.0560 (2)	0.0512 (13)	
C3	0.4200 (5)	0.2864 (3)	0.0996 (3)	0.0567 (14)	
H3	0.3505	0.3211	0.0888	0.068*	
C4	0.4409 (5)	0.2704 (3)	0.1594 (3)	0.0595 (15)	
H4	0.3856	0.2946	0.1874	0.071*	
C5	0.5434 (5)	0.2189 (3)	0.1765 (2)	0.0528 (13)	
H5	0.5580	0.2087	0.2159	0.063*	
C6	0.6269 (5)	0.1813 (3)	0.1329 (2)	0.0423 (11)	
C7	0.7364 (5)	0.1284 (3)	0.1531 (2)	0.0459 (12)	
H7	0.7519	0.1223	0.1930	0.055*	
C8	1.0000	0.0000	0.1068 (3)	0.0467 (17)	
C9	0.4052 (7)	0.3261 (4)	−0.0244 (3)	0.0779 (19)	
H9A	0.4081	0.3283	−0.0663	0.117*	
H9B	0.4405	0.3729	−0.0086	0.117*	
H9C	0.3093	0.3185	−0.0118	0.117*	
O4	0.579 (2)	0.1913 (12)	0.3182 (9)	0.098 (9)	0.234 (11)
H1M	0.5601	0.1599	0.3453	0.148*	0.234 (11)
C10	0.703 (3)	0.2337 (18)	0.3207 (10)	0.075 (10)	0.234 (11)
H10A	0.7221	0.2638	0.3554	0.113*	0.234 (11)
H10B	0.7221	0.2638	0.2861	0.113*	0.234 (11)
H10C	0.7632	0.1890	0.3207	0.113*	0.234 (11)
H1O	0.743 (5)	0.134 (3)	0.048 (2)	0.057 (15)*	
H2N	0.916 (5)	0.040 (3)	0.183 (3)	0.052 (15)*	

### Atomic displacement parameters (Å^2^)

**Table:**

	*U*^11^	*U*^22^	*U*^33^	*U*^12^	*U*^13^	*U*^23^
O1	0.062 (2)	0.055 (2)	0.051 (2)	0.0187 (18)	−0.0048 (18)	−0.0067 (17)
O2	0.077 (4)	0.069 (3)	0.047 (3)	0.019 (3)	0.000	0.000
O3	0.076 (3)	0.063 (2)	0.066 (3)	0.026 (2)	−0.0196 (19)	−0.001 (2)
N1	0.043 (2)	0.045 (2)	0.053 (2)	0.004 (2)	−0.0026 (19)	0.0038 (19)
N2	0.054 (3)	0.059 (2)	0.047 (3)	0.020 (2)	−0.001 (2)	0.004 (2)
C1	0.038 (3)	0.044 (2)	0.056 (3)	0.000 (2)	−0.002 (2)	−0.005 (2)
C2	0.046 (3)	0.042 (2)	0.065 (3)	0.004 (2)	−0.010 (3)	0.001 (3)
C3	0.039 (3)	0.049 (3)	0.082 (4)	0.006 (2)	−0.001 (3)	−0.005 (3)
C4	0.047 (3)	0.052 (3)	0.080 (4)	0.002 (3)	0.017 (3)	−0.008 (3)
C5	0.046 (3)	0.053 (3)	0.059 (3)	−0.002 (2)	0.014 (2)	0.002 (3)
C6	0.039 (3)	0.035 (2)	0.052 (3)	−0.001 (2)	0.004 (2)	−0.002 (2)
C7	0.047 (3)	0.044 (3)	0.046 (3)	−0.001 (2)	0.000 (2)	0.006 (2)
C8	0.047 (4)	0.040 (4)	0.053 (5)	0.003 (3)	0.000	0.000
C9	0.082 (4)	0.058 (3)	0.094 (5)	0.015 (3)	−0.025 (4)	0.014 (3)
O4	0.117 (19)	0.087 (15)	0.091 (17)	0.004 (12)	0.030 (13)	0.010 (12)
C10	0.07 (2)	0.10 (3)	0.057 (16)	0.021 (19)	−0.001 (11)	−0.018 (15)

### Geometric parameters (Å, º)

**Table:**

O1—C1	1.389 (6)	C4—H4	0.9300
O1—H1O	0.91 (5)	C5—C6	1.433 (7)
O2—C8	1.243 (8)	C5—H5	0.9300
O3—C2	1.397 (6)	C6—C7	1.466 (6)
O3—C9	1.445 (6)	C7—H7	0.9300
N1—C7	1.305 (6)	C8—N2^i^	1.381 (6)
N1—N2	1.381 (5)	C9—H9A	0.9600
N2—C8	1.381 (6)	C9—H9B	0.9600
N2—H2N	0.94 (6)	C9—H9C	0.9600
C1—C2	1.413 (6)	O4—C10	1.39 (3)
C1—C6	1.418 (7)	O4—H1M	0.8500
C2—C3	1.405 (8)	C10—C10^ii^	1.06 (5)
C3—C4	1.411 (8)	C10—H10A	0.9700
C3—H3	0.9300	C10—H10B	0.9700
C4—C5	1.382 (7)	C10—H10C	0.9700
			
C1—O1—H1O	102 (3)	N1—C7—C6	121.0 (4)
C2—O3—C9	118.1 (4)	N1—C7—H7	119.5
C7—N1—N2	113.9 (4)	C6—C7—H7	119.5
N1—N2—C8	119.1 (5)	O2—C8—N2	125.4 (3)
N1—N2—H2N	120 (3)	O2—C8—N2^i^	125.4 (3)
C8—N2—H2N	121 (3)	N2—C8—N2^i^	109.2 (7)
O1—C1—C2	116.1 (5)	O3—C9—H9A	109.5
O1—C1—C6	123.9 (4)	O3—C9—H9B	109.5
C2—C1—C6	120.0 (5)	H9A—C9—H9B	109.5
O3—C2—C3	126.5 (5)	O3—C9—H9C	109.5
O3—C2—C1	114.8 (5)	H9A—C9—H9C	109.5
C3—C2—C1	118.7 (5)	H9B—C9—H9C	109.5
C2—C3—C4	121.7 (5)	C10—O4—H1M	119.9
C2—C3—H3	119.2	C10^ii^—C10—O4	177.6 (17)
C4—C3—H3	119.2	C10^ii^—C10—H10A	63.1
C5—C4—C3	120.2 (5)	O4—C10—H10A	118.9
C5—C4—H4	119.9	C10^ii^—C10—H10B	63.1
C3—C4—H4	119.9	O4—C10—H10B	114.5
C4—C5—C6	119.4 (5)	H10A—C10—H10B	109.6
C4—C5—H5	120.3	C10^ii^—C10—H10C	87.1
C6—C5—H5	120.3	O4—C10—H10C	93.3
C1—C6—C5	120.1 (4)	H10A—C10—H10C	109.6
C1—C6—C7	122.3 (4)	H10B—C10—H10C	109.6
C5—C6—C7	117.5 (4)		

Symmetry codes: (i) −*x*+2, −*y*, *z*; (ii) −*x*+3/2, −*y*+1/2, *z*.

### Hydrogen-bond geometry (Å, º)

**Table:**

*D*—H···*A*	*D*—H	H···*A*	*D*···*A*	*D*—H···*A*
O1—H1*O*···N1	0.91 (5)	1.86 (5)	2.703 (5)	152 (5)
N2—H2*N*···O3^iii^	0.94 (6)	2.38 (5)	3.044 (6)	128 (4)
N2—H2*N*···O1^iii^	0.94 (6)	2.33 (6)	3.204 (6)	155 (4)

Symmetry code: (iii) *x*+1/4, −*y*+1/4, *z*+1/4.

## References

[bb1] Bacchi, A., Carcelli, M., Pelagatti, P., Pelizzi, C., Pelizzi, G. & Zani, F. (1999). *J. Inorg. Biochem.* **75**, 123–133. 10.1016/s0162-0134(99)00045-8 10450607

[bb2] Du, L. & Zhang, W. (2010). *Acta Cryst.* E**66**, o2645. 10.1107/S1600536810037517 PMC2983342 21587616

[bb3] El-Gammal, O. A., Abu El-Reash, G. M., Ghazy, S. E. & Radwan, A. H. (2012). *J. Mol. Struct.* **1020**, 6–15.

[bb4] Enraf–Nonius (1994). *CAD-4 EXPRESS* Enraf–Nonius, Delft, The Netherlands.

[bb5] Farrugia, L. J. (2012). *J. Appl. Cryst.* **45**, 849–854.

[bb6] Harms, K. & Wocadlo, S. (1995). *XCAD4* University of Marburg, Germany.

[bb7] He, Q.-P., Tan, B. & Lu, Z.-H. (2010). *Acta Cryst.* E**66**, o2968. 10.1107/S1600536810043151 PMC3009339 21589135

[bb8] Kong, L., Qiao, Y., Gao, Z. & Ju, X. (2010). *Acta Cryst.* E**66**, o2901. 10.1107/S1600536810042121 PMC3009061 21589078

[bb9] Sheldrick, G. M. (2008). *Acta Cryst.* A**64**, 112–122. 10.1107/S0108767307043930 18156677

